# *lon* Deletion Impairs Persister Cell Resuscitation in Escherichia coli

**DOI:** 10.1128/mbio.02187-21

**Published:** 2022-01-18

**Authors:** Sayed Golam Mohiuddin, Aslan Massahi, Mehmet A. Orman

**Affiliations:** a Department of Chemical and Biomolecular Engineering, University of Houston, Houston, Texas, USA; University of Texas Southwestern Medical Center Dallas

**Keywords:** *Escherichia coli*, Lon protease, ofloxacin, persisters, resuscitation, Z-ring

## Abstract

Bacterial persisters are nongrowing cells highly tolerant to bactericidal antibiotics. However, this tolerance is reversible and not mediated by heritable genetic changes. Lon, an ATP-dependent protease, has repeatedly been shown to play a critical role in fluoroquinolone persistence in Escherichia coli. Although *lon* deletion (Δ*lon*) is thought to eliminate persister cells via accumulation of the cell division inhibitor protein SulA, the exact mechanism underlying this phenomenon is not yet elucidated. Here, we show that Lon is an important regulatory protein for the resuscitation of the fluoroquinolone persisters in E. coli, and *lon* deletion impairs the ability of persister cells to form colonies during recovery through a *sulA-* and *ftsZ-*dependent mechanism. Notably, this observed “viable but nonculturable” state of antibiotic-tolerant Δ*lon* cells is transient, as environmental conditions, such as starvation, can restore their culturability. Our data further indicate that starvation-induced SulA degradation or expression of Lon during recovery facilitates Z-ring formation in Δ*lon* persisters, and Z-ring architecture is important for persister resuscitation in both wild-type and Δ*lon* strains. Our in-depth image analysis clearly shows that the ratio of cell length to number of FtsZ rings for each intact ofloxacin-treated cell predicts the probability of resuscitation and, hence, can be used as a potential biomarker for persisters.

## INTRODUCTION

Persisters are a small subset of cells within an isogenic cell population whose tolerance to antibiotics is transient and not mediated by genetic mutations. Persister cells significantly contribute to the crisis of antibiotic failure, as they can promote the evolution of antibiotic-resistant mutants (1–3) and the recurrence of microbial infections (4). The phenotypic state of persister cells is reversible, and their formation and resuscitation mechanisms can be triggered by stochastic and/or environmental factors (5–10).

The ATP-dependent Lon protease is perhaps one of the most well-studied bacterial proteases (11–15). Lon can degrade a wide range of cellular proteins, including regulatory proteins, such as SulA (16–18), RscA (19), and TER (20), as well as misfolded proteins (21), and can also act as a chaperone to prevent protein aggregation (21, 22). Deletion of *lon* has been frequently shown to reduce fluoroquinolone persistence (11, 12), suggesting that this protein may be an attractive target for small molecular inhibitors (23). However, the proposed mechanisms underlying the *lon*-dependent persistence state are highly controversial (14, 24). Lon was initially thought to induce persistence by degrading antitoxin molecules through a ppGpp/polyphosphate-dependent mechanism, but this model is no longer supported, as the reported observations were due to artifacts from a notorious laboratory contaminant (25). Another suggested mechanism for the role of Lon in bacterial persistence depends on the cell division inhibitor protein SulA and is based on the observation that deletion of *sulA* restores fluoroquinolone persister levels in *lon*-deficient strains (12, 26). In this model, accumulation of SulA in the absence of Lon should inhibit FtsZ-dependent ring formation in fluoroquinolone-treated persister cells (12, 26–28), thus impairing persister cell resuscitation and colony formation. Notably, this hypothesis, which remains unverified, may contradict the notion of persister cell dormancy. In other words, persister cells may not respond to fluoroquinolones or express SulA due to their dormant state. However, two independent groups analyzed the SOS response in ofloxacin-treated Escherichia coli cultures at single-cell resolution (29, 30) and found that antibiotic-induced DNA damage is similar in both persisters and antibiotic-sensitive cells. These findings demonstrate that persister cells can respond to external factors and indicate the presence of unique physiological activities in these cells that may be essential for their survival and resuscitation.

Studies of persisters are based on the premise that if a proposed mechanism is essential for the persister phenotype, genetically perturbing that mechanism should eliminate or reduce persister abundance. Such cells are quantified by persistence assays (e.g., clonogenic survival assays) in which culture samples are collected at various intervals during antibiotic treatment, washed, and plated on standard growth medium to enumerate surviving cells that can colonize in the absence of antibiotics (31). Unfortunately, this standard method does not distinguish persister formation mechanisms from resuscitation mechanisms. Thus, it is unclear from previous studies whether targeting Lon protease chemically or genetically eradicates persister cells or simply converts them to a viable but nonculturable (VBNC) state. To address this question, in the current study, we used vector constructs that allow fine-tuning of recombinant protein expression to verify that *lon* deletion (Δ*lon*) impairs the resuscitation of ofloxacin (OFX) persisters by inhibiting FtsZ-dependent ring formation. We further showed that the reduction of persisters among *lon*-deficient cells can be transient based on environmental conditions, such as starvation. Indeed, starvation-induced SulA degradation or expression of Lon during the recovery period (i.e., after removal of antibiotics) restores the ability of nonculturable Δ*lon* cells to form colonies by facilitating Z-ring formation, which represents a potential biomarker for Δ*lon* persister cells transitioning to the normal cell state.

## RESULTS

### Lon is required for resuscitation of fluoroquinolone persisters.

**FIG 1:**
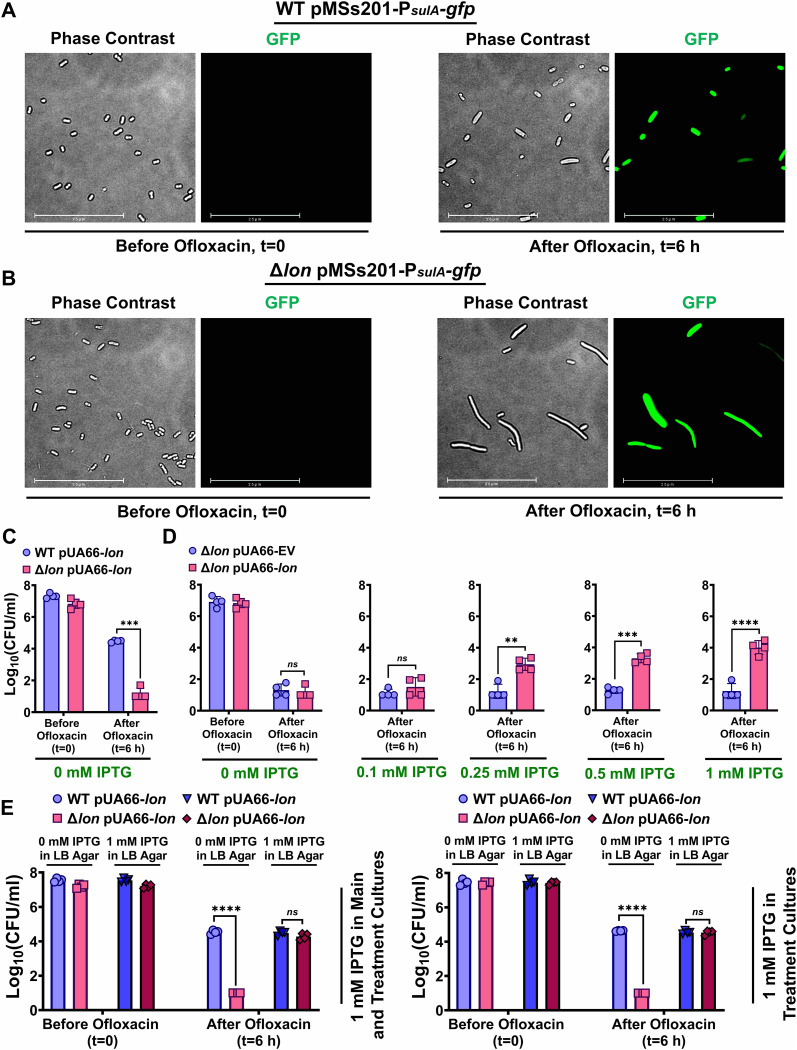
Lon overexpression rescues colony formation in OFX-treated Δ*lon* cells. (A and B) WT E. coli MG1655 (A) and Δ*lon* E. coli MG1655 (B) containing the *sulA-gfp* reporter plasmid (pMSs201-P*_sulA_-gfp*) were grown to stationary phase and diluted 100-fold in fresh LB medium. Diluted cells were then treated with 5 μg/mL OFX for 6 h. After treatment, phase-contrast and fluorescence (GFP) micrographs of cells were obtained. Images at 0 (*t* = 0) and 6 h (*t* = 6 h) from a representative replicate are shown, but similar data were obtained from four biological replicates (*n* = 4). Scale bar, 25 μm. (C and D) E. coli cells (WT and Δ*lon*) containing an inducible *lon* overexpression plasmid (pUA66-*lon*) or empty vector control (pUA66-EV) were treated with OFX (5 μg/mL) for 6 h and transferred to LB agar plates supplemented with isopropyl β-d-1-thiogalactopyranoside (IPTG) at the indicated concentrations. CFU were measured for each strain before and after treatment. *n* = 4. (E) WT and Δ*lon* E. coli containing pUA66-*lon* were cultured with 1 mM IPTG before and/or during OFX treatment. After treatment, cells were transferred to agar plates with or without 1 mM IPTG. CFU were measured for each strain before and after treatment. *n* = 4. Statistical significance for pairwise comparisons was assessed using one-way analysis of variance (ANOVA) with Dunnett’s *post hoc* test. **, *P* < 0.01; ***, *P* < 0.001; ****, *P* < 0.0001; ns, nonsignificant.

Fluoroquinolone antibiotics such as OFX inhibit DNA gyrase, leading to formation of double-stranded DNA breaks and induction of DNA repair mechanisms in persister cells (29, 30, 32). Consistent with previous studies (11, 26), we found that Δ*lon* in E. coli MG1655 cells also significantly reduces levels of persisters compared to those found in a wild-type (WT) strain in cell cultures treated with OFX for 6 h (Fig. S1A in the supplemental material). While deletion of *sulA* from the WT strain (Δ*sulA*) did not affect OFX persister levels in E. coli, its deletion from the Δ*lon* strain (Δ*lon*Δ*sulA*) restored persister levels of the *lon*-deficient cells (Fig. S1B), further verifying the reproducibility of previous studies (11, 26). Using a *sulA* reporter (pMSs201-P*_sulA_-gfp*) in which the *sulA* promoter (P*_sulA_*) is fused to a gene encoding green fluorescent protein (GFP) (33), we assessed *sulA* expression in both WT and Δ*lon* cells after OFX treatment (Fig. 1A and B). Although we identified a subset of OFX-treated cells that could not express GFP, we detected a significant number of GFP-positive cells expressing *sulA* in both the WT and Δ*lon* strains, with increased filamentation observed in *lon*-deficient cells, an expected morphological feature mediated by SulA accumulation (16, 34) (Fig. 1A and B).

10.1128/mBio.02187-21.1 FIG S1 Lon overexpression rescues colony formation in OFX-treated Δ*lon* cells. (A and B) Stationary phase cells of E. coli MG1655 WT, Δ*lon*, Δ*sulA*, and Δ*sulA*Δ*lon* strains were diluted 100-fold in fresh medium and then treated with OFX (5 μg/mL) for 6 h. At indicated time points, cells were collected, washed, serially diluted, and spotted on agar plates to enumerate persister cells. Number of biological replicates, *n* = 4 (A) and *n* = 3 (B). (C) Exponential-phase cells (E. coli MG1655 WT and Δ*lon* strain) were treated with OFX (5 μg/mL) for 6 h. At indicated time points, cells were washed and transferred to agar plates to enumerate persister cells (*n* = 4). (D and E) E. coli cells (the WT or the Δ*lon* strains) containing an inducible *lon* overexpression plasmid (pUA66-*lon*) or the empty vector (pUA66-EV) were treated with OFX (5 μg/mL) at exponential phase for 6 h and then transferred to agar plates supplemented with IPTG at the indicated concentrations. CFU were measured for each strain before and after treatment (*n* = 4). *F* statistics were used to compare kill curves. Statistical significance for pairwise comparison was assessed using one-way analysis of variance (ANOVA) with Dunnett’s *post hoc* test. ***, *P* < 0.001; ****, *P* < 0.0001; ns, nonsignificant. Download FIG S1, TIF file, 2.8 MB. Copyright © 2022 Mohiuddin et al. 2022 Mohiuddin et al. https://creativecommons.org/licenses/by/4.0/ This content is distributed under the terms of the Creative Commons Attribution 4.0 International license.

To determine whether increased accumulation of SulA converts Δ*lon* persisters to VBNC cells, we overexpressed Lon protease from a low-copy-number plasmid in *lon-*deficient cells during the recovery period on agar plates. This plasmid expression system was constructed using a cassette containing the isopropyl β-d-1-thiogalactopyranoside (IPTG)-inducible T5 promoter, *lon*, and the strong *LacI^q^* repressor, which was integrated into a pUA66 plasmid variant, thereby generating pUA66-*lon*. An empty vector (EV) without *lon* and a noninduced condition with pUA66-*lon* served as controls. Stationary-phase Δ*lon* cells harboring either pUA66-*lon* or EV were diluted in fresh medium and treated with OFX for 6 h. Lon expression was not induced before or during treatment. OFX-treated cells were then collected, washed with sterile phosphate-buffered saline (PBS) solution to remove the antibiotic, and spotted onto Luria-Bertani (LB) agar plates containing different concentrations of IPTG to induce *lon* expression. We found that Lon overexpression during recovery rescues growth of OFX persisters in a concentration-dependent manner; this increase in colony-forming ability was not observed in the absence of IPTG or with the EV control (Fig. 1C and D). The same phenomenon was also observed in cell populations obtained from exponential-phase cultures (Fig. S1C to E). We verified that presence of the plasmid-based expression systems does not significantly alter the levels of persister cells observed in wild-type (WT) and Δ*lon* strains (Fig. S2A). In addition, overexpressing Lon in OFX-treated WT cells during recovery did not significantly affect WT persistence (Fig. S2A). Next, to determine the role of Lon in E. coli persistence before and/or during OFX treatment, we added IPTG to cultures before and/or during treatment. Treated cells were then transferred to agar plates with or without IPTG for recovery (Fig. 1E). Although adding IPTG, and, thus, induction of Lon, at different stages did not impact WT persistence, Δ*lon* persisters could only be rescued when IPTG was added in LB agar plates during the recovery period (Fig. 1E and Fig. S2B). These data further highlight the importance of Lon in OFX persister resuscitation.

10.1128/mBio.02187-21.2 FIG S2 Expression vectors do not affect the WT persister levels. (A) Stationary-phase cells of indicated strains were diluted 100-fold in fresh liquid medium and then treated with OFX (5 μg/mL) for 6 h. After the treatment, the cells were collected, washed, serially diluted, and spotted on agar plates supplemented with or without IPTG (1 mM) for the persister cell enumeration (*n* = 3). (B) Cells of the indicated strains were cultured with 1 mM IPTG before and/or during OFX treatment. After the treatment, the cells were transferred to agar plates with or without 1 mM IPTG. CFU were measured for each strain before and after the treatment. (*n* = 4). Dashed lines indicate the limit of detection. Statistical significance for pairwise comparison was assessed using one-way analysis of variance (ANOVA) with Dunnett’s post-hoc test. **, *P* < 0.01; ***, P < 0.001; ns, nonsignificant. Download FIG S2, TIF file, 2.8 MB. Copyright © 2022 Mohiuddin et al. 2022 Mohiuddin et al. https://creativecommons.org/licenses/by/4.0/ This content is distributed under the terms of the Creative Commons Attribution 4.0 International license.

### SulA induction in the absence of OFX reduces culturability of the Δ*lon* strain.

**FIG 2:**
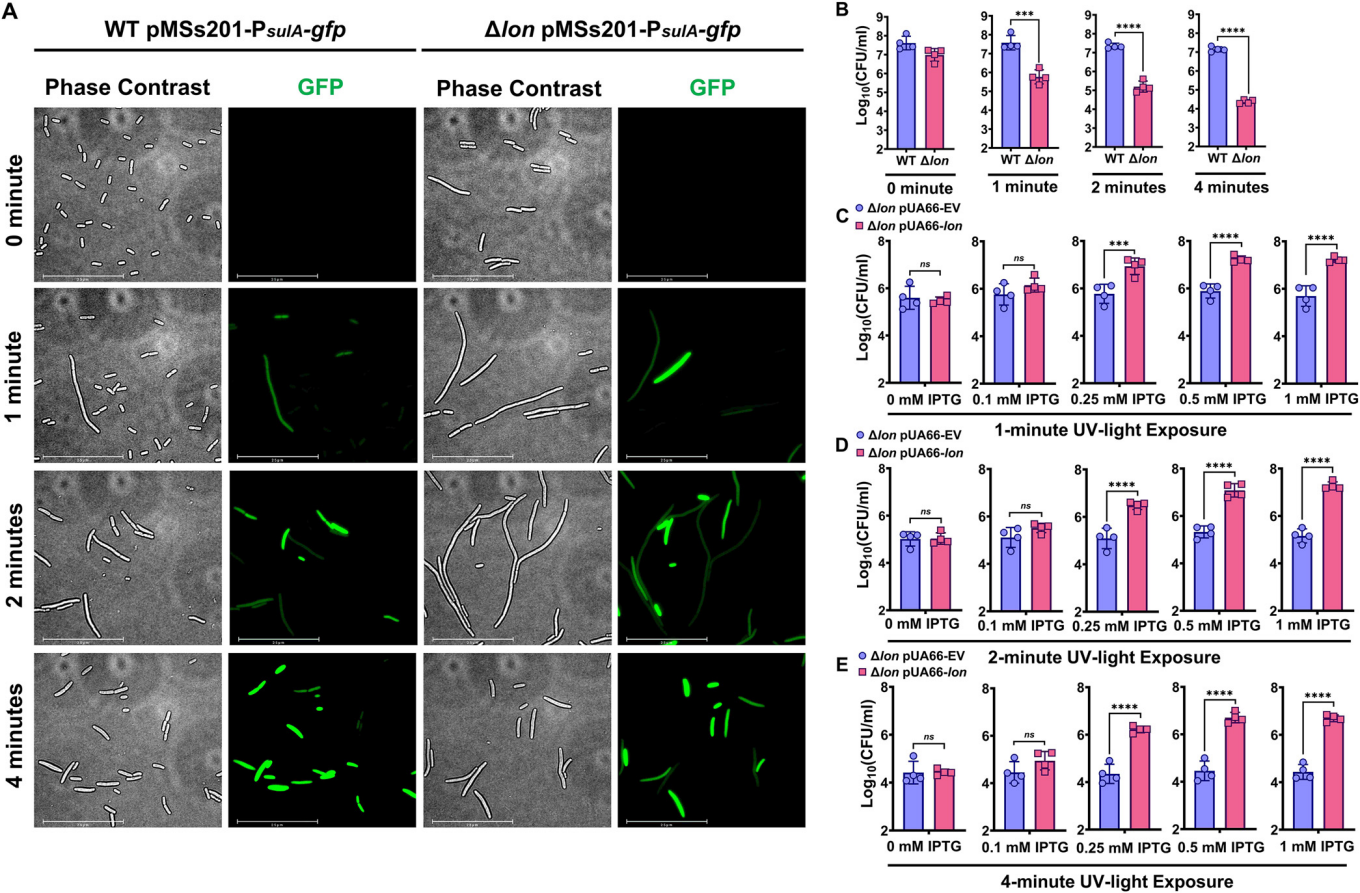
Lon overexpression rescues the colony-forming ability of Δ*lon* cells exposed to UV. (A) WT and Δ*lon* E. coli containing pMSs201-P*_sulA_-gfp* were grown to stationary phase, diluted 100-fold in fresh LB medium, and exposed to UV for the indicated times. After exposure, cells were cultured in a shaker for 2 h, and phase-contrast and fluorescence (GFP) micrographs of cells were obtained. Images from a representative replicate are shown, but similar data were obtained for four biological replicates. *n *= 4. Scale bar, 25 μm. (B) WT and Δ*lon* E. coli were exposed to UV as described in panel A and spotted onto LB agar plates to enumerate CFU. *n* = 4. (C to E) Cells containing the inducible *lon* overexpression plasmid (pUA66-*lon*) or empty vector (pUA66-EV) control were exposed to UV for the indicated times and transferred to agar plates supplemented with IPTG at indicated concentrations to enumerate CFU. *n* = 4. Statistical significance for pairwise comparisons was assessed using one-way ANOVA with Dunnett’s *post hoc* test. ***, *P* < 0.001; ****, *P* < 0.0001; ns, nonsignificant.

Given that exposure to UV light induces both DNA damage and expression of SulA in both WT and Δ*lon* strains (35, 36), we next tested whether the fluoroquinolone-mediated phenomenon described above also occurs in cells exposed to UV in the absence of OFX. To this end, we diluted stationary-phase cells in fresh medium, exposed these diluted cells to UV light for various time intervals, and then plated the cells on solid medium to determine total CFU. Cells harboring the *P_sulA_-gfp* reporter were also subjected to UV exposure, cultured in liquid medium for 2 h, and examined microscopically to assess SulA expression. We found that similar to OFX treatment, UV exposure induced *sulA* expression in both WT and Δ*lon* strains and increased filamentation of Δ*lon* cells (Fig. 2A). Further, whereas our chosen time intervals of UV exposure did not affect WT cell viability or CFU levels (Fig. 2B), in response to this treatment, cells lacking Lon displayed significantly lower CFU than WT cells in a UV exposure time-dependent manner (Fig. 2B). Next, we tested whether Lon overexpression could rescue this colony formation deficiency. Following UV exposure, Δ*lon* cells containing pUA66-*lon* or EV were plated on solid agar medium containing different concentrations of IPTG. As expected, we found that Lon expression eliminated the observed reduction in the culturability of Δ*lon* cells in response to UV (Fig. 2C to E). We further detected a positive correlation between IPTG (*lon* inducer) and CFU levels (Fig. 2C to E), suggesting that recovery is dependent on Lon protein concentration, as well as on levels of accumulated SulA.

Both chemical (OFX) and environmental (UV) induction of SulA attenuated the colony-forming ability of *lon*-deficient cells (Fig. 1 and 2), suggesting this phenomenon should be reproduced by plasmid-mediated SulA overexpression in the Δ*lon* strain. To accomplish this, we generated a plasmid (pBAD-*sulA*) expressing *sulA* under the arabinose-inducible P*_BAD_* promoter and transferred this plasmid into both WT and Δ*lon* strains harboring pUA66-*lon* or EV. We first noticed that leaky expression from pBAD-*sulA* inhibited growth of Δ*lon* cells in liquid pretreatment cultures (Fig. S3A). Therefore, we supplemented both WT and Δ*lon* pretreatment cultures with 0.25 mM IPTG to maintain Δ*lon* cell growth. Both WT and Δl*on* strains harboring the plasmids were grown to stationary phase, washed to remove inducer, and then plated on solid medium containing arabinose (*sulA* inducer) and/or IPTG (*lon* inducer) at various concentrations to differentially express SulA and/or Lon during colony formation. Notably, although we did not fine-tune expression levels of these proteins on plates, we observed reduced CFU of WT cells and almost no CFU of the Δ*lon* strain (under the limit of detection) at higher arabinose concentrations (>10 mM) (Fig. S3A and B). Further, whereas lower arabinose concentrations (<5 mM) did not affect the colony-forming ability of WT cells, colony formation of the Δ*lon* strain was significantly compromised (Fig. S3B) but reversed upon addition of IPTG (Fig. S3C), similar to what we observed for cells exposed to OFX and UV (Fig. 1 and 2).

10.1128/mBio.02187-21.3 FIG S3 SulA overexpression in the absence of OFX reduces the culturability of the Δ*lon* strain. (A) Stationary-phase cells harboring Lon and SulA overexpression plasmids were spotted on agar plates supplemented with arabinose at indicated concentrations for the CFU enumeration. Lon and SulA were not induced in precultures (*n* = 4). (B) Stationary-phase cells harboring Lon and SulA overexpression plasmids were spotted on agar plates supplemented with arabinose at indicated concentrations. IPTG (0.25 mM) was added in precultures to induce Lon (*n* = 4). (C) Stationary-phase cells harboring Lon and SulA overexpression plasmids were spotted on agar plates supplemented with IPTG or/and arabinose at indicated concentrations. IPTG (0.25 mM) was added in precultures to induce Lon (*n* = 4). Dashed lines indicate the limit of detection. Pairwise statistical significance was performed using one-way ANOVA with Dunnett’s posttest. *, *P* < 0.05; ****, *P* < 0.0001; ns, nonsignificant. Download FIG S3, TIF file, 2.9 MB. Copyright © 2022 Mohiuddin et al. 2022 Mohiuddin et al. https://creativecommons.org/licenses/by/4.0/ This content is distributed under the terms of the Creative Commons Attribution 4.0 International license.

### Starvation rescues Δ*lon* persisters.

**FIG 3:**
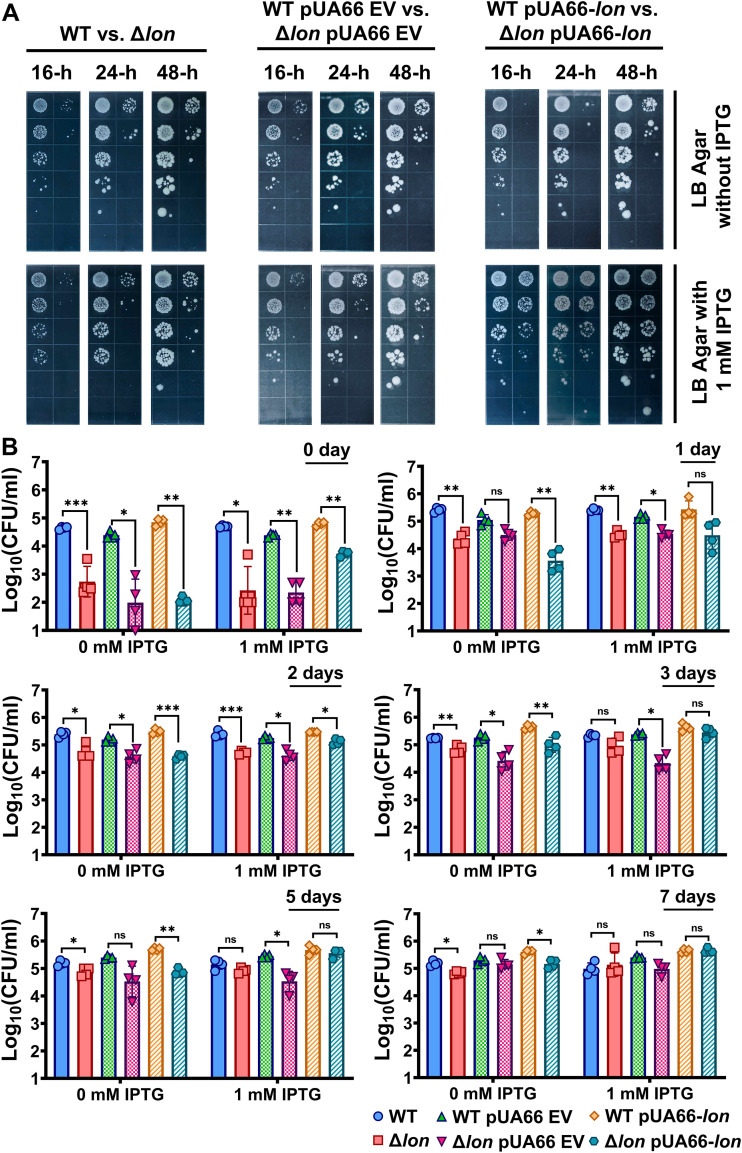
Starvation in PBS solution rescues Δ*lon* persisters. (A) WT and Δ*lon* E. coli with or without the inducible *lon* overexpression plasmid (pUA66-*lon*) or empty vector control (pUA66-EV) were grown to stationary phase and diluted 100-fold in fresh LB medium. Diluted cells were then treated with OFX for 6 h. After treatment, cells were washed, serially diluted, and spotted onto LB agar plates with or without IPTG (1 mM). Plates were incubated for 48 h, and images were captured at indicated time points. A representative replicate is shown, but similar data were obtained for four biological replicates. *n* = 4. (B) WT and Δ*lon* E. coli with or without pUA66-*lon* or pUA66-EV were treated with OFX for 6 h, transferred to sterile PBS solution with or without 1 mM IPTG, and incubated at 37°C in a shaker for 7 days. Samples were collected at the indicated time points and spotted onto agar plates to enumerate surviving cells. *n* = 4. Statistical significance for pairwise comparison was assessed using one-way ANOVA with Dunnett’s post hoc test. *, *P* < 0.05; **, *P* < 0.01; ***, *P* < 0.001; ns, nonsignificant.

Persister cells are typically quantified using clonogenic survival assays in which antibiotic-treated cells are plated on agar medium and then incubated for at least 16 h. Therefore, we next tested whether a longer incubation period is necessary for Δ*lon* colony formation (Fig. 3A). We found that, although a small number of both WT and Δ*lon* colonies emerged at later time points and that existing colonies grew larger with longer incubation times, the 2- to 3-log difference in CFU between the WT and Δ*lon* strains did not change with longer incubations (Fig. 3A). We made a similar observation for strains harboring pUA66-*lon* or EV that were plated on agar medium lacking IPTG (Fig. 3A). Thus, while IPTG-induced Lon during recovery on agar medium could rescue Δ*lon* cells harboring pUA66-*lon* (Fig. 3A), it was not clear whether Δ*lon* cells that could not form colonies in the absence of IPTG (Fig. 3A) were truly alive. To further investigate the ability of Δ*lon* cells to form colonies and assess the resilience of Δ*lon* persisters, cells with or without expression vectors were transferred to PBS solution after OFX treatment (37, 38), and their colony-forming ability was tested daily by plating samples on agar medium with or without IPTG for 7 days (Fig. 3B). We found that persister subpopulations from both WT and Δ*lon* strains were alive and able to survive for at least the 7-day duration of the experiment. Surprisingly, Δ*lon* cells, even those without any expression vectors, could be gradually resuscitated when incubated in PBS solution (Fig. 3B).

**FIG 4:**
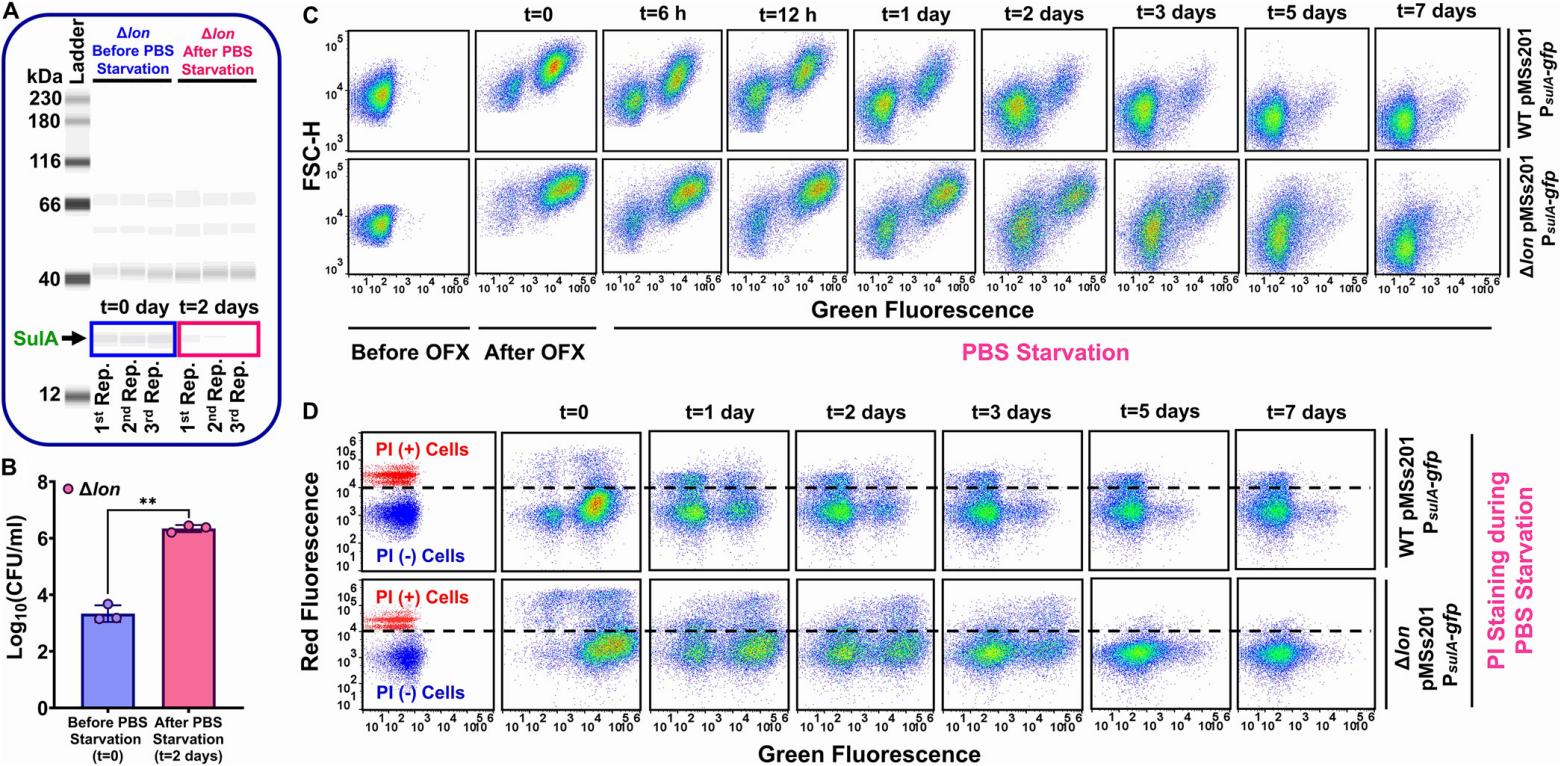
Starvation enhances cellular protein degradation. (A and B) Stationary-phase cells of the Δ*lon* strain were diluted 10-fold in fresh LB medium and treated with OFX for 6 h. Treated cells were collected, washed, and transferred to sterile PBS solution and cultured at 37°C in shaker for 2 days. Western blotting was performed at indicated time points (*t* = 0 and 2 days) to measure SulA expression (19 kDa). CFU counts were obtained before and after PBS incubation. *n* = 3. (C) The WT and Δ*lon* strains with pMSs201-P*_sulA_-gfp* were transferred to PBS solution after OFX treatment and cultured at 37°C with shaking for 7 days. Cells were analyzed with a flow cytometer for GFP measurements at indicated time points. A representative replicate is shown, but similar data were obtained for four biological replicates. *n* = 4. (D) OFX-treated cells described in panel C were stained with propidium iodide (PI) and analyzed with a flow cytometer. Live cells and ethanol (70% [vol/vol])-treated cells (dead cells) served as negative and positive controls, respectively. A representative replicate is shown, but similar data were obtained for four biological replicates. *n* = 4. Pairwise statistical significance was performed using two-tailed *t* test with unequal variance, where **, *P* < 0.01.

Given that SulA can be targeted by proteases other than Lon (39) and that starvation may further enhance its intracellular degradation (38, 40, 41), we measured SulA concentrations in OFX-treated Δ*lon* cell populations (including dead, VBNC, and persister cells) at days *t* = 0 and *t* = 2 during incubation in PBS solution (Fig. 4A) and confirmed a decrease in SulA levels over time. Because persister cells were scarce, we used high-cell-density cultures for persister cell enrichment, which were generated using a procedure (see Materials and Methods) that does not eliminate the observed phenotypic switch during starvation but increases the number of resuscitated persister cells (Fig. 4B). To test if protein degradation is a general characteristic of starved cells, we used WT and Δ*lon* cells harboring pMSs201-P*_sulA_-gfp* and monitored GFP levels in OFX-treated cells during starvation. Although the antibiotic induced GFP expression from the *sulA* promoter, cellular GFP started to decrease when the OFX-treated cells were starved in PBS solution (Fig. 4C). Notably, we identified a subset of cells that did not respond to OFX or express GFP, consistent with our data in Fig. 1A and B. These cells might have been dead residual cells and/or VBNC cells; their condition remains to be determined. To show that the observed GFP reduction is not solely attributed to leakage of the proteins through damaged membranes, we stained the cells with propidium iodide (PI), which does not permeate cells with intact membranes. PI staining verified that a significant number of intact WT and Δ*lon* cells still exhibited increased GFP degradation after starvation (Fig. 4D). Also, we observed an enrichment of intact cells at later time points of PBS incubation, as some dead cells were eventually lysed during PBS incubation (Fig. 4D). Altogether, our results imply that the resuscitation of Δ*lon* cells under starvation conditions might result from increased SulA degradation.

### PBS starvation facilitates persister resuscitation in both WT and *Δlon* recovery cultures.

**FIG 5:**
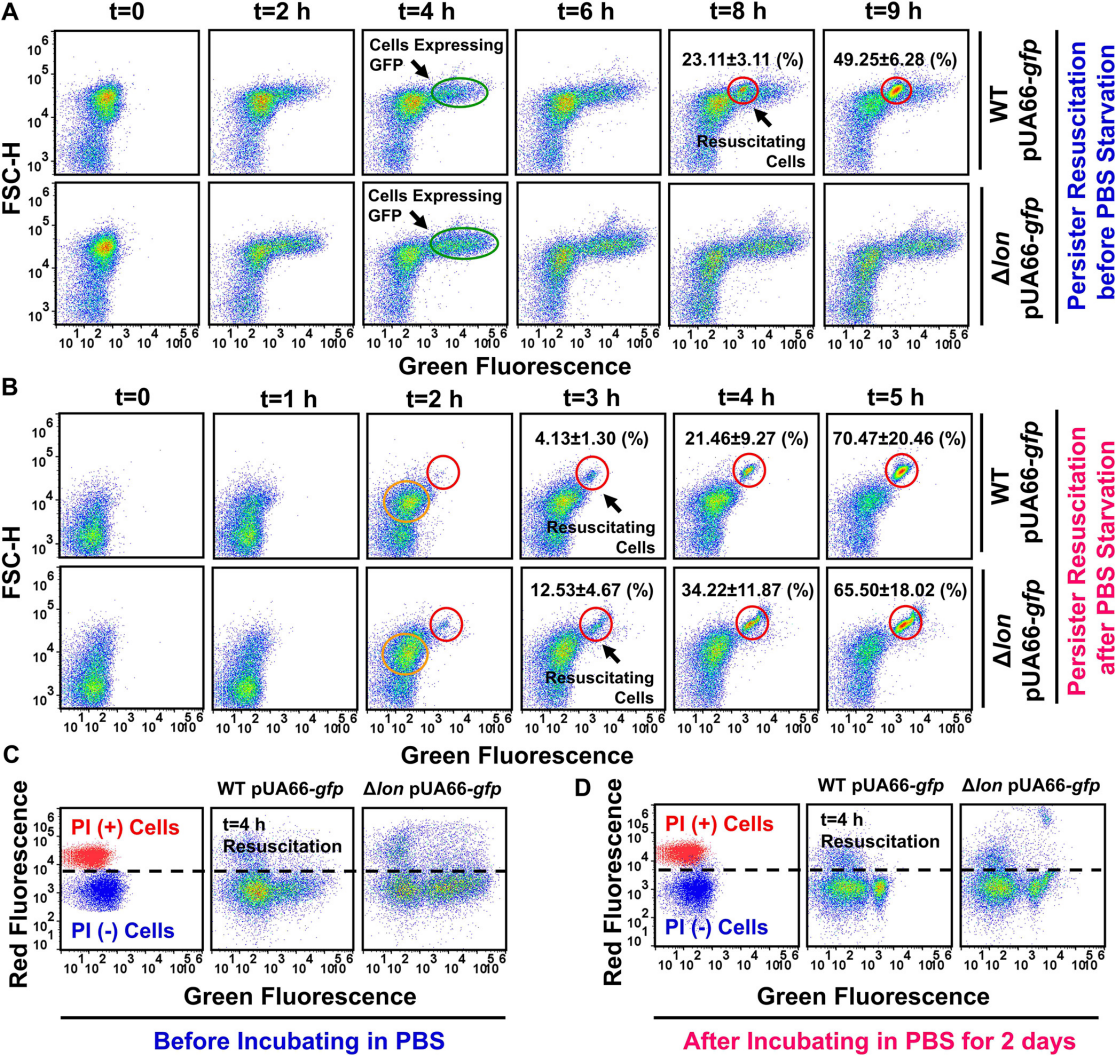
PBS starvation facilitates persister resuscitation in both WT and *Δlon* recovery cultures. (A) WT and Δ*lon* containing an IPTG-inducible *gfp* expression plasmid (pUA66-*gfp*) were transferred to fresh LB recovery medium containing 1 mM IPTG after 6 h of OFX treatment. IPTG was not added to cultures before or during treatment. At designated time points during recovery, cells were collected and analyzed with a flow cytometer. (B) WT and Δ*lon* cells containing pUA66-*gfp* were transferred to PBS solution after 6 h of OFX treatment and cultured at 37°C with shaking for 2 days. Cells were then collected and resuspended in fresh LB recovery medium and grown in the presence of 1 mM IPTG. At designated time points, cells were collected from the recovery culture and analyzed with a flow cytometer. (C and D) Cells described in panels A and B, respectively, were stained with PI at the indicated time point and analyzed with a flow cytometer. Live cells and ethanol (70% [vol/vol])-treated cells (dead cells) served as negative and positive controls, respectively. Proliferating cell fractions in the recovery populations are highlighted on the flow cytometry diagrams. A representative biological replicate is shown here. All replicates produced consistent results. *n* = 4.

Given that Δ*lon* potentially converts persisters to VBNC cells, we employed flow cytometry and an IPTG-inducible *gfp* expression system (pUA66-*gfp*) to monitor persister cell resuscitation. After OFX treatment, the WT and Δ*lon* strains harboring pUA66-*gfp* were transferred to fresh LB recovery medium (liquid) containing IPTG. We note that GFP was not induced in pretreatment cultures or during OFX treatment. When persister cells are resuscitated and then proliferate in recovery cultures, the cells should express GFP in the presence of IPTG and be detectable with flow cytometry (42). Interestingly, our analysis revealed that a large number of OFX-treated cells in both WT and Δ*lon* recovery cultures started to express GFP (Fig. 5A, subpopulations highlighted with green circles) while preserving their membrane integrity (Fig. 5C). Although the GFP-expressing WT cells in recovery cultures corresponded to ∼3.28 ± 0.53% of the initial cell population (before OFX treatment), the percentage of these cells was much higher in the Δ*lon* recovery cultures (∼7.31 ± 1.46%). Given that these cells can express GFP and that only a small fraction of them (i.e., persisters) could exit the nongrowth state and proliferate upon their transition to fresh medium (WT persisters, 0.125 ± 0.032% of the initial population; Δ*lon* persisters, ∼0.0015 ± 0.001% of the initial population), we presume that these GFP-positive cells largely exhibit VBNC phenotypes. The proliferating subpopulation in the WT recovery culture became more noticeable upon flow cytometry at approximately 7 to 8 h after transfer to new medium (Fig. 5A, subpopulation highlighted with a red circle), whereas this subpopulation was not detected in the Δ*lon* recovery culture throughout the course of the study (Fig. 5A). However, when starved in PBS solution for 2 days, Δ*lon* persisters could be resuscitated in recovery cultures similar to WT cells (Fig. 5B, subpopulations highlighted with a red circle). Although we did not observe many GFP-expressing cells after starvation, the recovery cultures still contained a significant number of intact cells (Fig. 5D) whose size started to increase upon their transfer to fresh medium, which was verified by their increased forward scatter (FSC-H) signals (Fig. 5B, subpopulations highlighted with orange circles). Although it is not clear whether these intact cells are truly VBNC cells (which is beyond the study’s scope), our data further showed that incubation in PBS solution facilitated persister resuscitation in both WT and Δ*lon* recovery cultures. Specifically, starved persister cells were resuscitated in less time (2 to 3 h) (Fig. 5B) than persisters from unstarved cell cultures (Fig. 5A). In addition, the resuscitating cells seemed to be elongated after starvation, and their size (i.e., FSC-H) considerably increased (Fig. 5B, subpopulations highlighted with red circles). Altogether, these results verify the existence of a phenotypic switch between a nonculturable to a culturable cell state in OFX-treated Δ*lon* cells that can be facilitated by starvation conditions.

### Starvation facilitates Z-ring formation in OFX-treated Δ*lon* cells.

Although SulA accumulation in *lon*-deficient cells is expected to inhibit Z-ring formation (28), this process has not been investigated in Δ*lon* persisters. Z-ring formation at the possible division site of a bacterium takes place when FtsZ, a filamentous tubulin-like protein, assembles into a ring shape (43–45). To study this event in Δ*lon* persisters, we generated and validated a low-copy expression system (pUA66-*ftsZ-gfp*) in which *ftsZ* is fused with *gfp* (46) and controlled by the IPTG-inducible T5 promoter (46). Here, using this FtsZ-GFP construct, we observed Z-ring formation in exponentially growing WT cells of various sizes and shapes, such as smaller cells with single rings and filamentous cells with randomly or orderly spaced multiple rings (data available upon request), as reported elsewhere (46–48). We also confirmed that FtsZ-GFP expression did not affect the observed 2- to 3-log difference between WT and Δ*lon* persistence (data available upon request).

**FIG 6:**
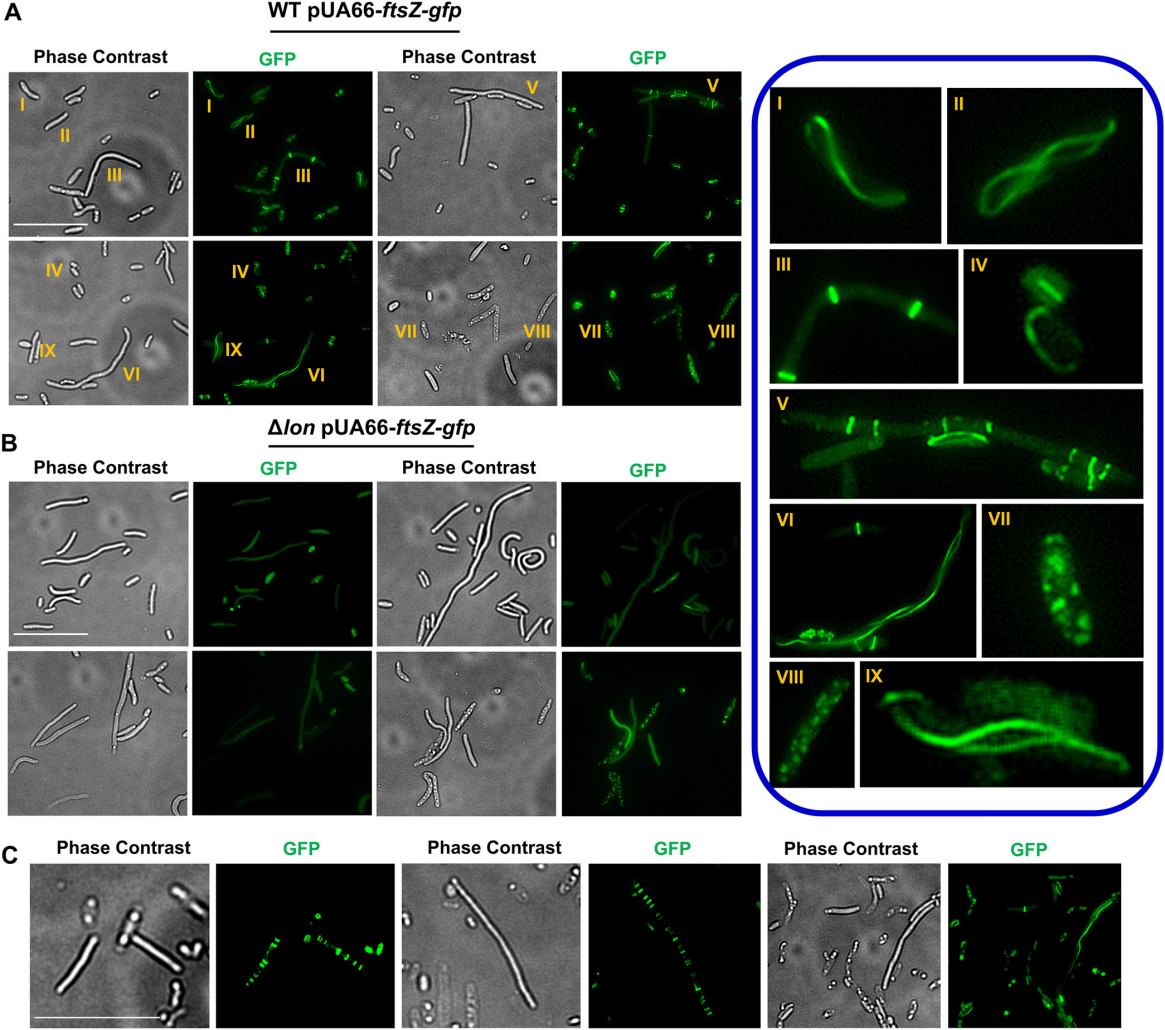
Starvation promotes Z-ring formation in OFX-treated Δ*lon* cells. (A and B) WT (A) and the Δ*lon* strain containing an IPTG-inducible FtsZ-GFP expression plasmid (pUA66*-ftsZ-gfp*) (B) were grown to stationary phase and diluted 100-fold in fresh LB medium. Diluted cells were then treated with OFX for 6 h. IPTG (1 mM) was added to cultures before and during treatment to express FtsZ-GFP. After treatment, cells were collected, washed with PBS solution to remove the antibiotic, and spread on an LB agarose (1%) pad. The pad was then monitored with a fluorescence microscope to collect phase-contrast and fluorescence images. The magnified FtsZ assemblies highlighted by numbers are provided in the right panel. (C) Δ*lon* cells harboring pUA66*-ftsZ-gfp* were transferred to PBS solution after OFX treatment and cultured for 2 days at 37°C with shaking (250 rpm). Then, cells were collected and transferred to pads to monitor FtsZ assemblies. Images from a representative replicate are shown, but similar data were obtained for four biological replicates. *n* = 4. Scale bar, 25 μm.

To investigate cellular Z-ring formation in OFX-treated cultures, cells harboring the FtsZ reporter were transferred to an LB agarose pad after OFX treatment and then monitored with fluorescence microscopy. OFX-treated WT cells showed highly heterogeneous cell sizes and morphologies, (e.g., smooth and rough cells) (Fig. 6A). FtsZ-GFP proteins were primarily aggregated in rough cells (Fig. 6A), which were potentially dead, as their membranes were highly damaged (Fig. S4A). FtsZ assemblies were generally found to be structurally heterogeneous in smooth, intact cells and included regularly spaced multiple Z-rings or linear, spiral, elliptical, and “8”-shaped structures that may have been transitioning to a ring shape (Fig. 6A and Fig. S4A). Similar to the WT strain, the heterogeneous FtsZ assemblies and cell morphologies were also observed in the Δ*sulA* and Δ*lon*Δ*sulA* strains (Fig. S5A, B). Conversely, Z-ring formation was rarely seen in Δ*lon* cell populations, which were instead enriched with cells in which FtsZ was dispersed in smooth cells or aggregated in rough cells (Fig. 6B and Fig. S4B). However, when starved in PBS solution for 2 days, OFX-treated Δ*lon* cultures formed cell subpopulations with structurally heterogeneous FtsZ assemblies, including randomly or orderly spaced multiple rings and linear or spiral filamentous structures (Fig. 6C). When we monitored OFX-treated WT, Δ*lon*, Δ*sulA*, and Δ*lon*Δ*sulA* cells on LB agar pads with time-lapse microscopy after starvation, we noted that healthy, elongated cells of all strains containing multiple highly organized Z-rings could be resuscitated within 2 to 4 h (Fig. S6). These cells first exhibited an extensive elongation, consistent with our flow cytometry data (Fig. 5B), and then divided at the septal points (49) (Fig. S6). Notably, we did not observe persister cells in which FtsZ polymerization underwent a structural change from linear or spiral to ring-shaped assemblies (44, 48). While shorter cells with fewer rings were rarely resuscitated, cells with aggregated proteins or dispersed GFP could not be resuscitated at all. Of note, protein aggregation was not due to the overexpression of FtsZ from the low-copy-number plasmids, we still observed aggregation phenotypes in OFX-treated cells without any expression vector in phase-contrast micrographs (data available upon request). Also, detection of resuscitating persisters in OFX-treated Δ*lon* cells with microscopy was extremely difficult when the cells were not starved in PBS.

10.1128/mBio.02187-21.4 FIG S4 Membranes of rough cells are highly damaged. (A and B) E. coli MG1655 WT (A) and Δ*lon* strains harboring the pUA66-*ftsZ-gfp* plasmid (B) were grown to stationary phase and diluted 100-fold in fresh medium; diluted cells were then treated with OFX for 6 h. IPTG (1 mM) was added in cultures before and during OFX treatment to express FtsZ-GFP. The treated cells were then collected, washed, and stained with PI for 15 minutes. The stained cells were washed with PBS to remove the dye and then spread on agarose (1%) pads. The pad was then monitored with a microscope to collect phase-contrast and fluorescent images. Representative images are shown; consistent images were obtained from all biological replicates (*n* = 3). Scale bar, 5 μm. Download FIG S4, TIF file, 2.7 MB. Copyright © 2022 Mohiuddin et al. 2022 Mohiuddin et al. https://creativecommons.org/licenses/by/4.0/ This content is distributed under the terms of the Creative Commons Attribution 4.0 International license.

10.1128/mBio.02187-21.5 FIG S5 The heterogeneous FtsZ assemblies and cell morphologies are observed in the Δ*sulA* and Δ*lon*Δ*sulA* strains. (A and B) E. coli MG1655 Δ*sulA* (A) and Δ*sulA*Δ*lon* strains containing the pUA66-*ftsZ-gfp* plasmid (B) were grown to stationary phase and diluted 100-fold in fresh medium; diluted cells were then treated with OFX for 6 h. IPTG (1mM) was added in cultures before and during OFX treatment to express FtsZ-GFP. After OFX treatment, the cells were collected, washed with PBS solution to remove the antibiotics, and spread on LB agarose (1%) pads. The pads were then monitored with a microscope to collect phase-contrast and fluorescent images. Representative images are shown; consistent images were obtained from all biological replicates (*n* = 3). Scale bar, 5 μm. Download FIG S5, TIF file, 2.8 MB. Copyright © 2022 Mohiuddin et al. 2022 Mohiuddin et al. https://creativecommons.org/licenses/by/4.0/ This content is distributed under the terms of the Creative Commons Attribution 4.0 International license.

10.1128/mBio.02187-21.6 FIG S6 Intact, elongated cells containing multiple highly organized Z-rings can be resuscitated within 2 to 4 h. E. coli MG1655 WT (A), Δ*sulA* (B), Δ*sulA*Δ*lon* (C), and Δ*lon* strains containing the pUA66-*ftsZ-gfp* plasmid (D) were transferred to PBS solution after 6 h OFX treatment and cultured at 37°C with shaking. IPTG (1mM) was added in cultures before, during, and after OFX treatment to express FtsZ-GFP. Cells incubated in PBS solution for 2 days were then collected, pelleted by centrifugation, and resuspended in LB medium and were then spread on LB agarose pads containing 1 mM IPTG. The pads were monitored with a fluorescence microscope having an onstage incubator. Arrows indicate the resuscitating cells whose fluorescent images are provided in the figure. Representative images are shown; consistent images were obtained from all biological replicates (*n* ≥ 3). Scale bar, 25 μm. Download FIG S6, TIF file, 2.9 MB. Copyright © 2022 Mohiuddin et al. 2022 Mohiuddin et al. https://creativecommons.org/licenses/by/4.0/ This content is distributed under the terms of the Creative Commons Attribution 4.0 International license.

### Z-ring architecture is a key biomarker of Δ*lon* persisters.

**FIG 7:**
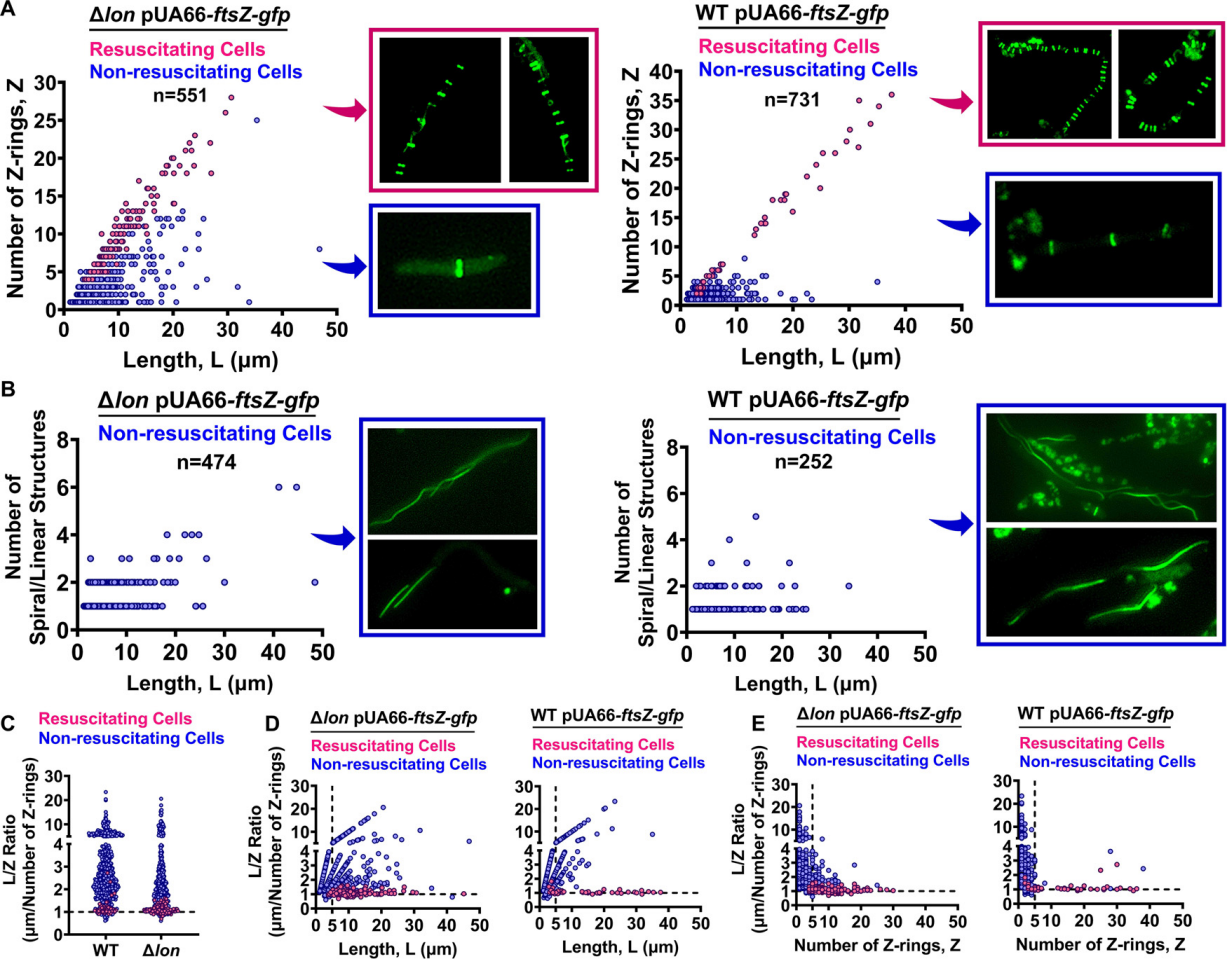
The number and structural organization of Z-rings represent a key persister biomarker. WT and Δ*lon* strains containing the IPTG-inducible FtsZ-GFP expression plasmid (pUA66-*ftsZ-gfp*) were transferred to PBS solution after 6 h of OFX treatment and cultured at 37°C with shaking. IPTG (1 mM) was added to cultures before and during OFX treatment to express FtsZ-GFP. Cells incubated in PBS solution for 2 days were then collected, pelleted by centrifugation, and resuspended in LB medium and were then spread on LB agarose pads containing 1 mM IPTG. The pads were monitored for 24 h with a fluorescence microscope with an onstage incubator. Cell lengths, as well as Z-ring structures and numbers, were determined for both resuscitating (persisters) and nonresuscitating (VBNC) cells to generate plots of the number of Z-rings versus cell length (A), the number of linear/spiral structures versus cell length (B), *L*/*Z* (cell length in μm/number of *Z* rings) ratios versus strain (C), *L*/*Z* versus cell length (D), and *L*/*Z* versus number of Z-rings (E). Pink color represents resuscitating cells, blue color represents nonresuscitating cells, and *n* represents the number of cells analyzed.

Although starvation facilitated persister resuscitation and Z-ring formation in the Δ*lon* strain, persister cells still represented only a small fraction of the intact cell subpopulation in antibiotic-treated cultures. To determine whether the Z-ring is a suitable biomarker for Δ*lon* persisters during their transition to a normal cell state, we monitored hundreds of intact but diverse (in cell shape, size, and Z-ring architecture) OFX-treated Δ*lon* cells with time-lapse microscopy after PBS starvation. Cell lengths, as well as Z-ring structures and numbers, were determined using phase-contrast and fluorescence microscopy directly after cells were transferred to microscope pads. Resuscitated cells were identified using time-lapse microscopy. Our in-depth image analysis revealed that persister resuscitation in the Δ*lon* strain strongly correlated with cell size and the number of Z-rings (Fig. 7A). Specifically, cells with linear or spiral FtsZ assemblies could not be resuscitated (Fig. 7B). Moreover, when we calculated the ratio of cell length in microns (*L*) to the number of Z-rings (*Z*) for each cell analyzed, we found a correlation between persister resuscitation and calculated *L*/*Z* values (Fig. 7C), which was supported by binomial regression analysis (Fig. S7). Cells with *L*/*Z* ≈ 1, *L* > 5 μm, and *Z* > 5 were generally resuscitated (Fig. 7D and E). While *L*/*Z* ratios of nonresuscitated cells were often much greater than 1 (Fig. 7C), we observed some nonresuscitating phenotypes in cells with *L*/Z ≈ 1. However, these cells were generally smaller (*L* < 5 μm) and had fewer Z-rings (*Z* < 5) than persister cells (Fig. 7D and E). Although we did not perform this labor-intensive analysis for the Δ*sulA* and Δ*lon*Δ*sulA* strains, we were able to report similar results for the WT strain (Fig. 7A to E), verifying the existence of a conserved relationship between persister resuscitation and Z-ring architecture in E. coli MG1655 under the conditions studied here.

10.1128/mBio.02187-21.7 FIG S7 Binomial logistic regression analysis. Graphs of logistic regression curves for E. coli WT and Δ*lon* strains showing the probability of resuscitation of a cell versus the cell length (L) (A and B), the probability of resuscitation of a cell versus the number of Z-rings (Z) (C and D), and the probability of resuscitation of a cell versus the L/Z ratio (E and F). Wald test *P* value was used to determine if the slope of the simple logistic model (β1) is significantly different from 0, which is equivalent to whether the odds ratio is 1.0. Red and blue dots represent cells that have been resuscitated (1) or not (0), respectively. Download FIG S7, TIF file, 2.8 MB. Copyright © 2022 Mohiuddin et al. 2022 Mohiuddin et al. https://creativecommons.org/licenses/by/4.0/ This content is distributed under the terms of the Creative Commons Attribution 4.0 International license.

The capability of persister cells to form highly organized multiple Z-rings (Fig. 7 and Fig. S6) should be enhanced by starvation (Fig. 5B). We have also noticed that *sulA* deletion induces thick and well-defined Z-ring structures in persister cells (Fig. S6). As the *sulA* promoter (P*_sulA_*) is tightly regulated by a RecA-LexA-dependent global DNA damage response mechanism, we presume that persisters (unlike VBNC cells) can successfully repair OFX-induced DNA damage, thus reducing SulA expression during the recovery period; this mechanism may explain the observed Z-ring structures in these cells (Fig. 7). Although the DNA repair mechanism(s) in persisters was not the main focus of this study, we analyzed P*_sulA_* activity of OFX-treated WT cells (harboring pMSs201-P*_sulA_-gfp*) in recovery cultures with fluorescence microscopy and flow cytometry. While most resuscitating WT cells were initially SulA positive after OFX treatment, their *sulA* promoter activity decreased when they began to elongate and divide in fresh, antibiotic-free medium (Fig. S8A). We also identified a cell subpopulation that continued to express SulA while elongating; however, these cells did not divide throughout the course of the study (Fig. S8A), implying the presence of DNA damage. Our flow cytometry analysis also showed that a large number of resuscitating cells had reduced *sulA* promoter activity (Fig. S8B). Although we did not monitor P*_sulA_* activity of OFX-treated cells after PBS starvation, as the GFP variant from pMSs201-P*_sulA_-gfp* was mostly degraded (Fig. 4C), the lack of DNA damage response in daughter cells highlights the ability of persister cells to efficiently repair OFX-induced cellular damage, which is consistent with a previously published study (29).

10.1128/mBio.02187-21.8 FIG S8 Resuscitating cells during recovery have reduced *sulA* promoter activity. WT E. coli MG1655 containing the pMSs201-P*_sulA_*-*gfp* plasmid were grown to stationary phase and diluted 100-fold in fresh medium; diluted cells were then treated with 5 μg/mL OFX for 6 h. (A) After the treatment, the cells were transferred to LB agar pads and monitored with a fluorescence microscope. (B) After OFX treatment, the cells were transferred to fresh LB recovery medium (liquid). The resuscitating cells (highlighted with red circles) in the recovery culture were determined with a flow cytometer. Representative images are shown; consistent images were obtained from all biological replicates (*n* = 3). Download FIG S8, TIF file, 2.9 MB. Copyright © 2022 Mohiuddin et al. 2022 Mohiuddin et al. https://creativecommons.org/licenses/by/4.0/ This content is distributed under the terms of the Creative Commons Attribution 4.0 International license.

### Lon overexpression during recovery facilitates Z-ring formation in OFX-treated Δ*lon* cells.

Finally, to demonstrate if Lon overexpression during recovery (without starvation) facilitates Z-ring formation in *lon*-deficient cells, we generated a pBAD-*ftsZ-gfp* plasmid (expressing the FtsZ reporter under the control of an arabinose-inducible promoter) and introduced it to the Δ*lon* strain harboring pUA66-*lon*. Because growth of the Δ*lon* strain with both expression vectors was markedly decreased on microscope pads, we performed resuscitation experiments in liquid cultures. Lon expression was not induced in cultures before and during antibiotic treatment. After OFX exposure, cells were transferred to fresh liquid medium with or without IPTG (the *lon* inducer), and at designated time points, samples from the resuscitation cultures were collected and plated on solid medium (with or without IPTG). We found that Δ*lon* cells started to resuscitate approximately 8 h after their transfer to fresh liquid medium with IPTG, as evidenced by increased CFU levels due to cell proliferation near the same time point (Fig. S9A). As expected, the resuscitation of Δ*lon* persisters was impaired in the absence of IPTG (Fig. S9A), and similar CFU profiles were obtained for Δ*lon* cells containing the vector control (Fig. S9A). We also investigated the collected samples with fluorescence microscopy and found that, despite their scarcity, persister cells began to form Z-rings after 8 h of culturing in the presence of IPTG and exhibited highly heterogeneous morphologies (Fig. S9B). FtsZ assemblies were also found to be structurally heterogeneous (e.g., linear and spiral structures) (Fig. S9B). Conversely, this phenomenon was rarely observed in Δ*lon* cultures in the absence of IPTG; rather, these cultures were enriched with nonresuscitating cells in which FtsZ was typically dispersed or aggregated (Fig. S9C).

10.1128/mBio.02187-21.9 FIG S9 Lon overexpression during recovery facilitates Z-ring formation in OFX-treated Δ*lon* cells. E. coli MG1655 Δ*lon* cells containing both pUA66-*lon* and pBAD*-ftZ-gfp* plasmids were treated with OFX for 6 h, transferred to fresh LB recovery medium (liquid) with or without IPTG (1 mM), and cultured in a shaker for persister cell recovery. Cells containing the empty vector served as controls. (A) At the indicated time points during recovery, cells were plated onto solid agar medium (with or without IPTG) to enumerate CFUs. (B and C) During persister cell resuscitation (8 to 12 h), cells were collected and spotted onto agarose pads (1%) to monitor Z-ring formation via fluorescence microscopy. Representative images are shown; consistent images were obtained from all biological replicates (*n* = 3). Scale bar, 5 μm. Download FIG S9, TIF file, 2.9 MB. Copyright © 2022 Mohiuddin et al. 2022 Mohiuddin et al. https://creativecommons.org/licenses/by/4.0/ This content is distributed under the terms of the Creative Commons Attribution 4.0 International license.

## DISCUSSION

The Lon protease has been shown to degrade misfolded proteins, RNases, heat shock proteins, and transfer-messenger RNA (tmRNA)-associated proteins, as well as components of chromosomal and/or plasmid-based toxin/antitoxin systems (16–22). Although these known functions and previously published data suggest Lon is involved in persister formation, our results have identified a crucial role for Lon in the resuscitation and recovery of persister cells. The work in this area, including the current study, suggests that SulA-Lon acts as a toxin-antitoxin module during quinolone treatment (SulA as a toxin) (11, 26). However, *sulA* only appears to be relevant in the absence of *lon*, as both the Δ*sulA* and double mutant Δ*lon*Δ*sulA* strains showed persister levels similar to those of WT cells. Nevertheless, Lon may still be an attractive target for small molecular inhibitors (23), as it is a critical protein for bacterial cell survival.

One way to differentiate persister formation mechanisms from those involved in persister resuscitation is to controllably express the genes of interest in their respective mutant strains before, during, and after antibiotic treatment. If a gene is essential for persister resuscitation, its expression in the mutant strain during recovery should be sufficient to restore the colony-forming ability of antibiotic-treated cells (29), as shown in this study, in which the inducible expression of Lon in Δ*lon* cells during recovery from OFX treatment increased their culturability. However, culturability of Δ*lon* persisters can also be restored when the cells are starved after OFX treatment. Interestingly, this phenomenon was also observed in WT cells, as we detected an increase (∼10-fold) in persisters of WT cultures during starvation (Fig. 3B). These results are consistent with those of previous studies (37, 50), which found that starvation, toxin induction, and chemical inhibition of transcription or translation following antibiotic treatment rescued E. coli persisters. Although these stressors delay growth-related processes which are thought to facilitate DNA repair and survival (37, 50), our data suggest that SulA degradation might also play a crucial role in persister rescue. However, these two proposed mechanisms are not mutually exclusive; in fact, they may occur in the cells simultaneously, as both are necessary for persister cell resuscitation.

We measured SulA concentrations in OFX-treated Δ*lon* cells, which included the entire cell populations of dead, VBNC, and persister cells. Unfortunately, direct measurement of persister cell physiology is challenging due to the difficulty in isolating pure samples. Many technical challenges arise from their low abundance, transient nature and similarities to VBNC cells, which are more abundant than persister cells. Given that (i) starvation enhances intracellular protein degradation (38, 40, 41), (ii) SulA is a fairly unstable protein (39), and (iii) cellular GFP was completely degraded in the entire cell population during incubation in PBS (Fig. 4), the resuscitation of Δ*lon* cells under starvation conditions potentially results from SulA degradation. This finding is further supported by our aforementioned experiments that show chemical (OFX) and environmental (UV) induction of SulA and plasmid-mediated SulA overexpression attenuate the colony-forming ability of *lon*-deficient cells.

One key insight from this study relates to issues associated with VBNC cells, which are generally more abundant than persister cells, can be metabolically active, stain as living cells, and preserve their membrane integrity (51). Further, although they also survive antibiotic treatments, their resuscitation in standard medium is typically not observed (52). In general, the clinical importance of VBNC cells of pathogenic bacteria is recognized, as certain bacteria (e.g., Mycobacterium tuberculosis) are known to survive in the human body for years without growing or causing symptoms (53, 54). These cells are fastidious under laboratory conditions and are most effectively detected and identified by PCR and immunological methods (54, 55). Critically, although perturbation of certain mechanisms may reduce persister levels in a cell population, VBNC cells may not be eliminated, which poses an important health concern. In this study, our data show that environmental signals can trigger VBNC cell resuscitation. Therefore, measuring VBNC cell levels in cultures is essential to better understand the mechanism by which they can resume growth. We previously developed flow cytometry techniques for this purpose (51, 56, 57) and showed here that resuscitation of VBNC Δ*lon* cells occurs under starvation conditions.

Another important finding from our study helps shed light on the long-standing unsolved question of why only a small fraction of intact cells can be resuscitated after the removal of antibiotics. Although microscopy, which has been used extensively by many research groups to study rare persister cells (30, 58, 59), can provide single-cell resolution, this approach cannot be reliably applied to distinguish persisters from VBNC cells without a persister-specific biomarker. This limitation is because both types of cells are in a growth-inhibited state during antibiotic treatment and can only be discriminated after persisters exit their persistence state and start to replicate following antibiotic removal. Resuscitation mechanisms may be regulated in a stochastic and threshold manner in persister cells during their transition to a normal cell state (58), but these mechanisms can only be elucidated if persister cells are detected and characterized during their transition and before they revert to normal, proliferating cells. Notably, our findings here suggest that Z-ring formation may be a key biomarker for distinguishing these cells.

Overall, the results of this study show that Lon plays a critical role in persister cell resuscitation through a mechanism dependent on SulA and FtsZ. We further demonstrate that the nonculturable state of antibiotic-tolerant Δ*lon* cells is transient, as their colony-forming ability can be restored by starvation, which likely leads to SulA degradation. Finally, we show that starvation-induced SulA degradation or expression of Lon during recovery facilitates Z-ring formation in Δ*lon* persisters, suggesting altered Z-ring architecture may be a biomarker for persister cells transitioning to a normal cell state.

## MATERIALS AND METHODS

### Bacterial strains and plasmids.

All experiments were conducted using E. coli MG1655 and its derivative strains. E. coli MG1655 and the pQE-80L and pUA66 plasmids were obtained from Mark P. Brynildsen at Princeton University, and pXY027 was a gift from Jie Xiao (plasmid no. 98915; Addgene, Watertown, MA, USA) (46). The Δ*lon*, Δ*sulA*, and Δ*lon*Δ*sulA* strains were generated using the Datsenko-Wanner method, as described previously (60). The pQE-80L plasmid has an isopropyl β-d-1-thiogalactopyranoside (IPTG)-inducible synthetic T5 promoter and a strong constitutive *LacI^q^* repressor. The pUA66*-gfp* plasmid variant was generated by transferring the T5-*gfp*-*lacI^q^* segment from pQE-80L*-gfp* into pUA66 that has kanamycin resistance gene (Kan^r^). Removal of *gfp* from pUA66*-gfp* led to development of a modified pUA66 empty vector. The *lon* gene was then cloned into the modified pUA66 plasmid to generate the pUA66-*lon* expression system. The SulA reporter (pMSs201-P*_sulA_-gfp*) was generated in a previous study (33). A pBAD plasmid variant containing the ampicillin resistance gene (Amp^r^), an arabinose-inducible promoter (P*_BAD_*), the *araC* gene encoding the P*_BAD_* inhibitor, and pBRR322*ori* were obtained from Thermo Fisher Scientific (catalog no. V44001; Waltham, MA, USA). The *sulA* gene was cloned into this pBAD plasmid to generate the pBAD-*sulA* expression system. To generate the pBAD-*ftsZ-gfp* expression system, a cassette containing the *ftsZ* gene fused with *gfp*, and a chloramphenicol resistance (Cm^r^) gene was amplified from pXY027 and ligated to the P*_BAD_*-*araC*-pBR322*ori* cassette obtained from the pBAD plasmid. To generate the pAU66-*ftsZ-gfp* plasmid, the *ftsZ-gfp* DNA fragment, amplified from pXY027 plasmid, was cloned into the modified pUA66 empty vector. FtsZ-GFP expression systems were transferred to WT, Δ*lon*, Δ*sulA*, and Δ*lon*Δ*sulA* strains that also contain the wild type *ftsZ* gene. All plasmids were generated using standard cloning methods (https://www.neb.com/tools-and-resources/feature-articles/foundations-of-molecular-cloning-past-present-and-future?__cf_chl_jschl_tk__=SNruurTMsKPXPp0X7YTcODunmJAHSq9MW9WNWyaasjM-1639784771-0-gaNycGzNCL0). Genetic modifications were verified by PCR and gene sequencing (Genewiz, South Plainfield, NJ, USA). A complete list of strains, plasmids, and oligonucleotides used in this study is presented in Table S1 in the supplemental material.

10.1128/mBio.02187-21.10 TABLE S1 Bacterial strains, plasmids, and oligonucleotides used in this study. Download Table S1, DOCX file, 0.02 MB. Copyright © 2022 Mohiuddin et al. 2022 Mohiuddin et al. https://creativecommons.org/licenses/by/4.0/ This content is distributed under the terms of the Creative Commons Attribution 4.0 International license.

### Media, chemicals, and culture conditions.

Unless stated otherwise, all chemicals, antibiotics, and enzymes used in these experiments were purchased from Thermo Fisher Scientific, VWR International (Radnor, PA, USA), Sigma-Aldrich (St. Louis, MO, USA), or New England Biolabs (Ipswich, MA, USA). All liquid media were prepared with ultrapure deionized (DI) water, having an electrical resistivity of 18.2 MΩ.cm. Luria-Bertani (LB) liquid medium was prepared by dissolving 10 g tryptone, 10 g sodium chloride, and 5 g yeast extract in 1 L ultrapure DI water. LB agar medium was prepared by dissolving 40 g premixed LB agar powder in 1 L ultrapure DI water. Phosphate-buffered saline (PBS, 1×) solution was used to remove chemicals and antibiotics from bacterial cell cultures; this was prepared by mixing 10× sterile PBS solution with autoclaved ultrapure DI water. Persister assays were performed with 5 μg/mL ofloxacin (OFX). The MIC of OFX for E. coli MG1655 was determined previously (56). To select for plasmid maintenance, kanamycin (25 μg/mL), chloramphenicol (25 μg/mL), and ampicillin (50 μg/mL) were added to both liquid and solid cultures. To induce expression of Lon, FtsZ, and SulA, medium was supplemented with the inducer (IPTG and l-arabinose, depending on the plasmid used; see Table S1 in the supplemental material for details) at the indicated concentrations. All chemicals and antibiotics added to bacterial cultures were dissolved in ultrapure DI water and sterilized by passage through 0.2-μm membrane filters. Solid and liquid media were sterilized by autoclaving. Overnight precultures and main cultures were prepared in test tubes containing 2-mL LB broth, and these were grown at 37°C with shaking (250 rpm) for 24 h. Overnight precultures were inoculated from cell stocks frozen in 25% glycerol at −80°C. Main cultures grown for 24 h were considered to be in stationary phase.

### Cell growth and persister assays.

To prepare main cultures, cells from overnight precultures were diluted 1:1,000 in 2-mL LB broth in 14-mL test tubes and cultured in a shaker for 24 h. Growth was assessed by measuring optical density at 600-nm wavelength (OD_600_) using a Varioskan Lux multimode microplate reader (Thermo Fisher Scientific). Stationary-phase cells from the main cultures (*t* = 24 h) were diluted 100-fold in 2-mL LB in 14-mL test tubes and treated with OFX (5 μg/mL) for 6 h. At the indicated time points, 1 mL of culture was removed from the test tubes, collected into microcentrifuge tubes, and centrifuged at 13,000 rpm to pellet the surviving cells. After centrifugation, 900 μL of supernatant was removed from the tubes, and 900 μL PBS solution was added. This washing procedure was repeated at least twice to reduce the antibiotic concentration to sub-MIC levels. After the final centrifugation, 900 μL of supernatant were removed; cell pellets were resuspended in the remaining 100 μL of PBS solution, and concentrated cell suspensions were transferred to the wells of a round-bottom 96-well plate. Cell suspensions were then serially diluted (10-fold) in PBS solution, and 10 μL of the diluted cell suspensions were spotted onto agar plates to enumerate persister levels. When required, cells were spotted onto agar plates supplemented with IPTG (0.1 to 1 mM), l-arabinose (1 to 20 mM), kanamycin (25 μg/mL), chloramphenicol (25 μg/mL), or ampicillin (50 μg/mL) at the indicated concentrations. To increase the limit of detection, the remaining 80 μL of the concentrated cell suspensions were plated. This serial dilution and plating procedure were also performed for cell cultures before antibiotic treatment to quantify the total number of cells in these cultures. To quantify persister cells in exponential-phase cultures, cells from an overnight culture were diluted (100-fold) in 25 mL LB in a 250-mL baffled flask, cultured until they reached an OD_600_ of ∼0.2, and then treated with OFX. At the indicated time points during treatment, cells were collected, washed, and plated to quantify CFU as described above. Plates were incubated at 37°C for at least 16 h to count CFU and then incubated up to an additional 48 h to see if new colonies emerged. Of note, we refer to overnight and main cultures as “pretreatment cultures” and OFX-treated cultures as “treatment cultures” in the manuscript. Solid and liquid cultures to which OFX-treated cells were transferred after removal of antibiotics are referred to as “recovery cultures.” When indicated, the Lon expression was induced in pre- and treatment cultures with 1 mM IPTG.

### UV exposure assay.

WT and Δ*lon* cells from stationary-phase cultures were diluted 1:100 in 1-mL LB, transferred to petri dishes, and exposed to UV light (UVP ChemStudio, catalog no. 849-97-0928-02; Analytik Jena, Jena, Germany) for different lengths of time (i.e., 0, 1, 2, and 4 min). After UV exposure, cells were collected, serially diluted in PBS solution, and then spotted onto agar plates (±1 mM IPTG). Agar plates were incubated for at least 16 h at 37°C to enumerate CFU. Cells expressing SulA reporters were similarly exposed to UV; these cells were then transferred to test tubes and cultured for 2 h in a shaker prior to imaging analysis (described below).

### SulA overexpression.

WT and Δ*lon* cells harboring the pBAD-*sulA* and pAU66-*lon* plasmids from stationary-phase cultures were serially diluted (10-fold) in PBS solution, and 10 μL of the diluted cell suspensions were spotted onto agar plates containing l-arabinose (the SulA inducer) at various concentrations (0, 1, 5, 10, and 20 mM) with/without 1 mM IPTG (the Lon inducer). Of note, pretreatment cultures of both WT and Δ*lon* strains were supplemented with 0.25 mM IPTG for Lon expression to maintain the growth of Δ*lon* cells, as the leaky expression of SulA from the pBAD-*sulA* plasmid impairs the growth of Δ*lon* cells in liquid pretreatment cultures.

### Starvation in PBS solution.

Stationary-phase WT and Δ*lon* cells were diluted 1:100 in 14-mL test tubes containing 2 mL LB and treated with OFX for 6 h. Treated cultures (2 mL) were collected, washed with PBS solution to remove antibiotics, resuspended in 2-mL sterile PBS solution, and cultured at 37°C with shaking (250 rpm) for 7 days. At the indicated time points (0, 1, 2, 3, 5, and 7 days), 100-μL cell samples were collected, serially diluted, and spotted onto agar plates (containing either 1-mM IPTG or no IPTG), as described above. Agar plates were incubated at 37°C for at least 16 h to enumerate CFU.

### Western blotting for SulA protein detection in high-cell-density cultures.

To quantify intracellular SulA protein levels, we used high-cell-density cultures. Briefly, stationary-phase cells were diluted 10-fold in 25 mL fresh LB medium in 250-mL flasks, treated with OFX for 6 h, and then washed with PBS solution to remove the antibiotic and resuspended in 25 mL PBS solution in flasks and cultured at 37°C with shaking (250 rpm). Cultures (including persister, VBNC, and dead cells) were collected into 50-mL falcon tubes on day 0 and day 2 during PBS starvation and centrifuged at 4,700 rpm for 15 min to sediment the cells, which were then frozen with dry ice and sent to RayBiotech (Peachtree Corners, GA, USA) for the SulA quantitation. Cells were also plated to quantify CFU. Cell lysis, protein quantitation, and detection were performed by RayBiotech scientists. Samples containing the same total protein concentration were analyzed by an automatic western machine, in which proteins are separated by size and immobilized in a capillary system. The target protein (SulA) was then chemiluminescently quantified using a primary antibody (rabbit anti-E. coli SulA polyclonal antibody; catalog no. MBS7004012; MyBioSource, San Diego, CA, USA) and a horseradish peroxidase-conjugated secondary antibody.

### Monitoring GFP degradation.

Stationary-phase cells of E. coli MG1655 WT and Δ*lon* strains harboring the pMSs201-P*_sulA_-gfp* plasmid were diluted 100-fold in 2-mL LB in 14-mL test tubes and treated with OFX (5 μg/mL) for 6 h. After the treatment, the cells were washed with PBS solution to remove the antibiotic, and resuspended in PBS solution and cultured at 37°C with shaking (250 rpm). Cells at indicated time points during the PBS incubation were analyzed with a flow cytometer (NovoCyte flow cytometer; NovoCyte 3000RYB; ACEA Biosciences Inc., San Diego, CA). Cells were excited at 488 nm, and green fluorescence was detected with a 530/30 nm emission filter. Live cells with or without GFP expression systems were used as controls.

### PI staining.

OFX-treated cells were collected, washed with PBS solution, and resuspended in 0.85% NaCl solution to achieve a desired cell density for flow cytometry analysis (∼10^6^ cells/mL). After adding PI (20 μM), cells were incubated in dark at 37°C for 15 min. The cells were then analyzed with a flow cytometer. Unstained live cells were used to gate the cell population on the flow cytometry diagram using forward and side scatter parameters. PI-stained dead cells obtained from ethanol (70%) treatment were used as a positive control. PI-stained live cells were used as a negative control. Cells were excited at 561-nm wavelength, and red fluorescence was detected with a 615/20-nm bandpass filter. When necessary, PI-stained cells were also analyzed with fluorescence microscopy (see “Microscope imaging” section for details).

### Monitoring persister cell resuscitation with flow cytometry.

OFX-treated WT and Δ*lon* cells harboring the pUA66-*gfp* plasmid were washed with PBS solution to remove the antibiotic and then resuspended in fresh LB medium containing 1 mM IPTG and cultured at 37°C with shaking (250 rpm). However, IPTG was not added in overnight and treatment cultures. At designated time points, cells were collected from the recovery culture and analyzed with a flow cytometer. Cells were excited at 488 nm, and green fluorescence was detected with a 530/30-nm emission filter.

### Microscope imaging.

### (i) Z-ring imaging.

Stationary-phase E. coli MG1655 WT, Δ*lon*, Δ*sulA*, and Δ*lon*Δ*sulA* cells harboring the pUA66-*ftsZ-gfp* plasmid were diluted 1:100-fold in 2-mL LB broth in 14-mL test tubes and then treated with OFX for 6 h. IPTG (1 mM) was added to overnight and main cultures to induce *ftsZ-gfp* expression. Treated cells were then collected, washed to remove the antibiotic, transferred to 2 mL PBS solution in 14-mL test tubes, and cultured at 37°C with shaking (250 rpm). After starving the cells in PBS solution for 2 days, 1-mL cell suspensions were collected from PBS cultures, pelleted by centrifugation, and resuspended in 1 mL LB media. The resuspended cells were then transferred to LB agar pads, which were dried next to a Bunsen burner flame for 30 min. The agar pads were prepared by dissolving agarose (1%) in LB liquid medium followed by microwave sterilization. After cooling the agarose medium, kanamycin (25 μg/mL) and IPTG (1 mM) were added for plasmid retention and *ftsZ-gfp* expression. The LB agarose medium was then poured over a glass slide (25 by 75 mm), each side of which was enclosed by stacked slides to make the pad smooth and sufficiently thick (61). Once the agar pads containing cells were dried, glass coverslips (25 by 75 by 0.17 mm) were placed on the top of the cells. The slides with the coverslips were also taped at the corners to make them stable enough for the microscope imaging. Agar pads were maintained in an onstage incubator (catalog no. AMC1000; Thermo Fisher Scientific) in which temperature and humidity were controlled during resuscitation and microscope imaging. Both phase-contrast and fluorescence (GFP) images were obtained using a fluorescence microscope (Evos FL Auto 2; catalog no. AMAFD2000; Thermo Fisher Scientific) with a 100× (oil) objective (Olympus, catalog no. AMEP4733; working distance, 0.3 mm) to determine cellular morphology and assess expression of the FtsZ-GFP fusion protein. An Evos GFP light cube (catalog no. AMEP4651) was used to acquire GFP images with 470/22-nm excitation and 510/42-nm emission wavelengths. To confirm that the pUA66-*ftsZ-gfp* plasmid can report the cellular ring structures, exponentially growing WT cells (harboring the reporter) that did not receive any treatment were monitored with microscopy as described above. The same microscopy procedure was also applied for the OFX-treated cells that were not starved.

### (ii) Imaging cells harboring the pUA66-*lon* and pBAD-ftsZ-*gfp* plasmids.

Stationary-phase WT or Δ*lon* cells containing the pUA66-*lon* and pBAD-ftsZ-*gfp* plasmids were diluted 1:100 in 2 mL LB broth in 14-mL test tubes and then treated with OFX for 6 h. Arabinose (1 mM) was added to overnight and main cultures to induce *ftsZ-gfp* expression. OFX-treated cells were collected, washed with PBS solution, and resuspended in 2 mL LB, supplemented with 10 mM arabinose (±1 mM IPTG) in 14-mL test tubes and grown in a shaker. At the indicated time points, one test tube was removed from the shaker; 1 mL of cell suspension was used for CFU enumeration, and the remaining 1 mL was used for the imaging process as described above.

### (iii) SulA imaging.

WT and Δ*lon* cells containing the *sulA* reporter plasmid (pMSs201-P*_sulA_-gfp*) were collected after OFX treatment or UV exposure, serially diluted, spotted onto agarose pads, and imaged with microscopy, as described above. When necessary, cells treated with OFX or exposed to UV were transferred to 1 mL PBS solution in 5-mL test tubes and analyzed via flow cytometry to quantify GFP-positive (i.e., SulA-expressing) cells. Cells were excited at 470/20 nm, and green fluorescence was detected with a 510/42-nm emission filter. Cells carrying the EV were used as controls.

### (iv) Image analysis.

To capture a heterogenous cell population in each replicate, 10 different locations in each agarose pad, leading to a total number of cells ranging from 200 to 1,000, were selected and monitored with the use of the Evos FL Auto 2 imaging system. Cell morphology and FtsZ ring structures in microscope images were analyzed with ImageJ software (62). Default ImageJ plugins and JavaScript (1.8.0) were used to process the images. The brightness of the phase-contrast images and the color of the fluorescent images were adjusted with “brightness/contrast,” “color balance,” and “sharpen” options. Given that dead, rough cells with aggregated proteins can be detected in both phase-contrast and fluorescent images, as shown in Fig. S4, we used phase-contrast images to investigate protein aggregation in the cells that do not have fluorescent protein expression systems. Cell lengths were measured manually using the segmented line tools in the ImageJ. The scale bars of raw images were used as a reference for the cell length measurements (62). Similarly, the number of Z-rings within a bacterium was enumerated manually using the fluorescent images.

### Statistical analysis.

At least three independent biological replicates were performed for each experiment. A nonlinear logarithmic model was used to generate biphasic kill curves (3, 56). *F* statistics were used to compare kill curves and to assess statistical significance (3, 56). Pairwise comparisons were performed using one-way analysis of variance (ANOVA) with Dunnett’s *post hoc* test or two-tailed *t* test with unequal variance. Each data point in the figures represents the mean value ± standard deviation. For microscopy analyses, representative images from both phase-contrast and fluorescence microscopy are displayed in the figures. The threshold values for significance were set at *, *P* < 0.05; **, *P* < 0.01; ***, *P* < 0.001; ****, *P* < 0.0001; and ns, nonsignificant. GraphPad Prism was used to generate the figures and to perform the statistical analyses.

Binary logistic regression analysis from GraphPad Prism was used to determine whether the probability of resuscitation of a cell depends on the cell length (*L*), the number of Z-rings within the cell (*Z*) or the *L*/*Z* ratio. The outcomes (resuscitation status) are encoded as 1 (indicating a “success” in resuscitation) or 0 (indicating a “failure” in resuscitation). The form of the simple logistic model is expressed as follows:

$$\text{log}\left(\text{odds}\right)={\text{β}}_{0}+{\text{β}}_{1}X$$

$$\text{odds}=\frac{p}{1-p}$$

where β_0_ and β_1_ are intercept and slope constants, respectively, and *p* is the probability of resuscitation of a cell. Wald test was used to determine if the slope of the simple logistic model (β_1_) is significantly different from 0, which is equivalent to whether the odds ratio is 1.0 (i.e., *P* = 1.0).
