# Supplementary material for: lon Deletion Impairs Persister Cell Resuscitation in Escherichia coli
Source: mBio. 2022 Jan 18;13(1):e02187-21. doi: 10.1128/mbio.02187-21 (PMC8764555; doi:10.1128/mbio.02187-21)
Supplement: TABLE S1 [file mbio.02187-21-st001.docx]

**SUPPLEMENTARY TABLE**

**Table S1: Bacterial strains, plasmids and oligonucleotides used in this study.**

| **Bacterial Strains** | | | | **Source or Reference** | |  |
| --- | --- | --- | --- | --- | --- | --- |
|  |  |  |  |  |  |  |
| *Escherichia coli* K-12 MG1655 Wild Type (WT) | | | |  | |  |
| *Escherichia coli* K-12 MG1655 Δ*lon* | | | | This study | |  |
| *Escherichia coli* K-12 MG1655 Δ*sulA* | | | | This study | |  |
| *Escherichia coli* K-12 MG1655 Δ*sulA* Δ*lon* | | | | This study | |  |
| **Plasmids** | | | | **Source or Reference** | |  |
| pMSs201 (*kan^R^*) | | | | Dharmacon Promoter Library | |  |
| pBAD/MycHisA (pBAD) (*amp^R^*) | | | | ThermoFisher Scientific | |  |
| pXY027 (*cm^R^*) | | | | Addgene | |  |
| pUA66-*gfp (kan^R^)* | | | | Brynildsen Lab | |  |
| pUA66*-ftsZ-gfp (kan^R^)* | | | | This study | |  |
| pQE-80L-*gfp (kan^R^)* | | | | Brynildsen Lab | |  |
| **Bacterial Strains with Plasmids** | | | | **Source or Reference** | |  |
| *Escherichia coli* K-12 MG1655 pMSs201-P*_sulA_-gfp* | | | | This study | |  |
| *Escherichia coli* K-12 MG1655 Δ*lon* pMSs201-P*_sulA_-gfp* | | | | This study | |  |
| *Escherichia coli* K-12 MG1655 WT pUA66-*EV* | | | | This study | |  |
| *Escherichia coli* K-12 MG1655 WT pUA66-*lon* | | | | This study | |  |
| *Escherichia coli* K-12 MG1655 Δ*lon* pUA66-*EV* | | | | This study | |  |
| *Escherichia coli* K-12 MG1655 Δ*lon* pUA66-*lon* | | | | This study | |  |
| *Escherichia coli* K-12 MG1655 WT pBAD*-ftsZ-gfp* | | | | This study | |  |
| *Escherichia coli* K-12 MG1655 Δ*lon* pBAD*-ftsZ-gfp* | | | | This study | |  |
| *Escherichia coli* K-12 MG1655 WT pUA66*-lon* pBAD*-ftsZ-gfp* | | | | This study | |  |
| *Escherichia coli* K-12 MG1655 *Δlon* pUA66*-lon* pBAD*-ftsZ-gfp* | | | | This study | |  |
| *Escherichia coli* K-12 MG1655 WT pUA66-*ftsZ-gfp* | | | | This study | |  |
| *Escherichia coli* K-12 MG1655 Δ*lon* pUA66-*ftsZ-gfp* | | | | This study | |  |
| *Escherichia coli* K-12 MG1655 Δ*sulA* pUA66-*ftsZ-gfp* | | | | This study | |  |
| *Escherichia coli* K-12 MG1655 Δ*sulA* Δ*lon* pUA66-*ftsZ-gfp* | | | | This study | |  |
| *Escherichia coli* K-12 MG1655 WT pUA66*-EV* pBAD*-EV* | | | | This study | |  |
| *Escherichia coli* K-12 MG1655 *Δlon* pUA66*-EV* pBAD*-EV* | | | | This study | |  |
| *Escherichia coli* K-12 MG1655 WT pUA66*-lon* pBAD*-sulA* | | | | This study | |  |
| *Escherichia coli* K-12 MG1655 *Δlon* pUA66*-lon* pBAD*-sulA* | | | | This study | |  |
| **Plasmid construction** | | | | | |  |
| pUA66-*gfp* | | The pUA66-*gfp* plasmid was previously generated in the Brynildsen Lab. The *T5-gfp-lacI^q^* DNA fragment was amplified from the pQE-80L-*gfp* plasmid, and then, cloned into the pUA66 backbone. Both the *T5-gfp-lacI^q^* fragment and the pUA66 plasmid were double-digested with AatII and SbfI restriction enzymes and, then, ligated. | | | |  |
| pUA66-EV  (empty vector) | | | A DNA fragment including *T5* promoter, *Kan^R^* gene, pUA66 origin of replication and *lacI^q^* was amplified from the pUA66-*gfp* plasmid with primers having BspHI cut sites. The amplified DNA fragment was digested with BspHI, and then self-ligated to obtain the modified pUA66-EV that does not have the *gfp* gene. | | | |
| pUA66-*lon* | | | The *lon* gene was amplified from the genomic DNA of *E. coli*, using forward and reverse primers with EcoRI and BglII restriction enzyme cut sites, respectively. The pUA66-*gfp* plasmid was double digested with EcoRI and BamHI to remove the *gfp* gene. Then, the digested *lon* gene and plasmid were ligated to generate pUA66-*lon*. | | | |
| pUA66*-ftsZ-gfp* | | | The *ftsZ-gfp* fragment and the pUA66 vector backbone were amplified from pXY027 and pUA66*-gfp* plasmids, using forward and reverse primers with BspHI and AatII restriction enzyme cut sites, respectively. Both the pUA66 backbone and the *ftsZ-gfp* fragment were double digested with the restriction enzymes, and then ligated to generate the pUA66*-ftsZ-gfp* plasmid. | | | |
| pBAD*-ftsZ-gfp* | | | *ftsZ-gfp-cm^R^* was amplified from the pXY027 plasmid, using primers with PciI cut sites. The pBAD plasmid was double digested with NcoI and BspHI restriction enzymes. Then, the digested PCR product and plasmid were ligated to generate pBAD-*ftsZ-gfp*. | | | |
| pBAD*-sulA* | | | The *sulA* gene was amplified from the genomic DNA of *E. coli*, using forward and reverse primers with PciI and EcoRI restriction enzyme cut site, respectively. The pBAD plasmid was double digested with NcoI and EcoRI enzymes. Then, the digested *sulA* gene and plasmid were ligated to generate pBAD-*sulA*. | | | |
| pMSs201-P*_sulA_-gfp* | | | Dharmacon Promoter Library | | | |
| **Oligonucleotides to generate gene deletions and plasmids construction** | | | | | | |
| **Plasmid/Deletion** | | **Forward Primer (5’ to 3’)** | | **Reverse Primer (5’ to 3’)** | | **Source** |
| Δ*lon*::*KAN^R^* | | ATCTGATTACCTGGCGGAAATTAAACTAAGAGAGAGCTCTGTGTAGGCTGGAGCTGCTTC | | TGCCAGCCCTGTTTTTATTAGTGCATTTTGCGCGAGGTCATTAACGGCTGACATGGGAAT | | Integrated DNA Technologies, Inc. |
| Δ*sulA*::*KAN^R^* | | CTGTACATCCATACAGTAACTCACAGGGGCTGGATTGATTGTGTAGGCTGGAGCTGCTTC | | TGGGCGACAAAAAAAGTTCCAGGATTAATCCTAAATTTACTTAACGGCTGACATGGGAAT | | Integrated DNA Technologies, Inc |
|  | |  | |  | |  |
| pUA66-*gfp* | | AGCTAGTTGACGTCATCGCTCGAGAAATCATAAAAAATTTAT | | GTCAGCATCCTGCAGGGCTAGACACCATCGAATGGTGCAAAACCT | | Integrated DNA Technologies, Inc. |
| pUA66-*EV* | | GCGCCTCATGAGGTACCCCGGGTCGACCTGCAGCCAAGCTTAATTA | | GCGCCTCATGAACTAGAGGTCTCCTCTTTAATGAATTCTGTGTG | | Integrated DNA Technologies, Inc. |
| pUA66-*lon* | | GCGCATGAATTCATTAAAGAGGAGACCTCTAGTatgAATCCTGAGCGTTCTGAACG | | GCGCTCAGATCTctaTTTTGCAGTCACAACCTGCAT | | Integrated DNA Technologies, Inc. |
| *ftsZ-gfp*  (pUA66*-ftsZ-gfp*) | | GCGCATGACGTCATTAAAGAGGAGACCTCTAGTATGTTTGAACCAATGGAACTTACC | | GCGCATTCATGATTATTTGTATAGTTCATCCATGCCATGTG | | Integrated DNA Technologies, Inc. |
| The pUA66 backbone  (pUA66*-ftsZ-gfp*) | | GCGCATTCATGAGCATGCGAGCTCGGTACCCCGGG | | GCGCATGACGTCTGTGTGAAATTGTTATCCGCTCACA | | Integrated DNA Technologies, Inc. |
| pBAD*-ftsZ-gfp* | | GCGCCACATGTTTGAACCAATGGAACTTACCAATGACG | | GCGCCACATGTGAAGCACACGGTCACACTG | | Integrated DNA Technologies, Inc. |
| pBAD*-sulA* | | GCGCCACATGTACACTTCAGGCTATGCACATCG | | GCGCCGAATTCttaATGATACAAATTAGAGTGAATTTTTAGCCCGG | | Integrated DNA Technologies, Inc. |
